# Association of NAFLD with FGF21 Polygenic Hazard Score, and Its Interaction with Protein Intake Level in Korean Adults

**DOI:** 10.3390/nu15102385

**Published:** 2023-05-19

**Authors:** Hae Jin Lee, Jinyoung Shon, Yoon Jung Park

**Affiliations:** 1 Department of Nutritional Science and Food Management, Ewha Womans University, Seoul 03760, Republic of Korea; 2 Graduate Program in System Health Science & Engineering, Ewha Womans University, Seoul 03760, Republic of Korea

**Keywords:** fibroblast growth factor 21 (FGF21), non-alcoholic fatty liver disease (NAFLD), polygenic hazard scores (PHS), protein intake, recommended nutrient intake (RNI)

## Abstract

Fibroblast growth factor 21 (FGF21) is a hormone that participates in the regulation of energy homeostasis and is induced by dietary protein restriction. Preclinical studies have suggested that FGF21 induction exerts a protective effect against non-alcoholic fatty liver disease (NAFLD), while human studies have revealed elevated levels of and potential resistance to FGF21 in patients with NAFLD. However, whether the FGF21 pathway also contributes to NAFLD risk at the genetic level remains uncertain. A few attempts to investigate the impact of individual genetic variants at the loci encoding FGF21 and its receptors on NAFLD risk have failed to establish a clear association due to a limited effect size. Therefore, this study aimed to (1) develop a polygenic hazard score (PHS) for FGF21-related loci that are associated with NAFLD risk and (2) investigate the effect of its interaction with protein intake level on NAFLD risk. Data on 3501 participants of the Korean Genome Epidemiology Study (Ansan–Ansung) were analyzed. Eight single-nucleotide polymorphisms of fibroblast growth factor receptors and beta-klotho were selected for PHS determination using forward stepwise analysis. The association between the PHS and NAFLD was validated (*p*-trend: 0.0171 for men and <0.0001 for women). Moreover, the association was significantly modulated by the protein intake level in all participants as well as women (*p*-interaction = 0.0189 and 0.0131, respectively) but not in men. In particular, the women with the lowest PHS values and a protein intake lower than the recommended nutrient intake (RNI) exhibited a greater NAFLD risk (HR = 2.021, *p*-trend = 0.0016) than those with an intake equal to or greater than the RNI; however, those with higher PHS values had a high risk, regardless of protein intake level. These findings demonstrate the contribution of FGF21-related genetic variants and restricted protein intake to NAFLD incidence.

## 1. Introduction

Non-alcoholic fatty liver disease (NAFLD) comprises a range of liver conditions caused by extra fat buildup in the liver without significant consumption of alcohol or lipid-causing drugs, viral infection, and/or inherited genetic diseases [1]. Its prevalence has been gradually increasing, currently affecting approximately 30% of the global adult population [2]. The prevention and treatment of NAFLD is an important public health concern, since NAFLD is closely associated with the risks of other metabolic disorders, including obesity, hyperlipidemia, and diabetes, and potentially leads to severe liver damage, such as cirrhosis or hepatocellular carcinoma [3]. Considering the tight association between metabolic dysregulation and fatty liver diseases, an attempt has recently been made to rename NAFLD to metabolic associated fatty liver disease (MAFLD) [4,5]. NAFLD often develops in people with obesity and diabetes; nevertheless, approximately 40% of patients with NAFLD in Korea are non-obese or lean [6]. Therefore, NAFLD is possibly not only attributed to metabolic stress-induced physiological dysfunction but also to other causes, such as genetic susceptibility [7]. Previous genome-wide association studies (GWAS) and candidate gene studies have identified genetic loci associated with NAFLD incidence, such as patatin-like phospholipase domain-containing 3 (*PNPLA3*), transmembrane 6 superfamily member 2 (*TM6SF2*), glucokinase regulator (*GCKR*), and lysophospholipase-like 1 (*LYPLAL1*) [8,9,10,11]. Most of these loci encode proteins that are directly involved in lipid metabolism, especially lipogenesis and cholesterol metabolism [7], exhibiting functional relevance.

The fibroblast growth factor 21 (FGF21) pathway is a metabolic pathway that has emerged as a promising target for therapeutic potentials against NAFLD [12]. FGF21, one of the fibroblast growth factor family members, is a stress-inducible hormone that functions in the regulation of metabolic homeostasis and energy balance [13,14,15]. It acts through binding to a heterodimeric receptor complex comprising beta-klotho and FGF receptors, including FGFR1, FGFR2, and FGFR3 [16,17,18,19]. The *FGF21* gene is widely expressed in metabolism-related organs, such as the liver, adipose tissue, and pancreas, while plasma FGF21 is mainly derived from the liver [20]. Pharmacological delivery of FGF21 has been reported to reduce hepatic fat accumulation [21,22] and demonstrate beneficial effects against obesity-related metabolic complications, including insulin resistance and hyperlipidemia [23,24]. However, plasma FGF21 levels are significantly higher in patients with NAFLD than in healthy individuals and are positively associated with the degree of the liver steatosis score in patients with NAFLD [25,26,27], indicating that the high-circulating FGF21 levels in patients with NAFLD does not appear to alleviate the condition; possibly due to resistance toward FGF21 [28]. This resistance highlights the potential importance of its receptors. The FGF21 signal is received predominantly by beta-klotho and FGF receptors. Alteration of receptor expression by alternative splicing and translational initiation potentially modulates FGF21 signaling [29,30]. Therefore, genetic or environmental factors possibly influence FGF21 expression, and its receptors may collectively contribute to the incidence and management of NAFLD.

Food-intake pattern, especially macronutrient distribution, is a key environmental factor for FGF21-pathway induction. Prolonged fasting is known to increase the expression and serum level of FGF21 [31,32,33]. The FGF21 response is likely due to protein restriction rather than energy restriction during fasting. The *FGF21*, *FGFR*, and *KLB* genes in the liver have been found to undergo upregulation upon isocaloric, low-protein feeding in animal models [34,35,36]. This phenomenon has also been observed in human intervention studies, as demonstrated by a meta-analysis on circulating FGF21 levels [37]. Previous animal experiments and nutritional intervention studies have also found restricted dietary protein intake to positively affect the transcriptional levels of FGF21 and its receptors [34,38,39]. Protein-restriction-stimulated FGF21 levels result in increased intracellular glucose uptake and energy consumption, possibly to compensate for the restriction [38,39], although the effects vary depending on the level of restriction [34,40]. Although FGF21 pathway-related macronutrients are various, studies dealing with the relation between protein and gene variant-related FGF21 are not enough. Therefore, we focused our attention on the protein intake rather than carbohydrate intake.

Currently, knowledge regarding the genetic contribution of FGF21 and its receptor loci to NAFLD risk remains limited. Only a few single-nucleotide polymorphisms (SNPs) at the FGF21 locus, such as rs838133, have had their associations with macronutrient preference and metabolic parameters investigated [41,42]. However, the results were based on cross-sectional cohorts and did not establish an association between FGF21 SNPs and NAFLD. Moreover, an approach based on individual SNPs has a limited effect size and power to analyze the association with disease risk and interactions with environmental factors [43].

Therefore, in this study, we hypothesized that genetic variations of the FGF21 pathway are collectively associated with NAFLD risk, with the associations potentially modulated by protein intake level, which is a strong FGF21-pathway stimulus. To investigate these associations, we (1) developed a polygenic hazard score (PHS) via the discovery and combination of multiple-loci SNPs related to the FGF21 pathway based on a longitudinal cohort of the Korean population, (2) analyzed the risk of NAFLD incidence based on the PHS, and (3) explored the possible modifying effect of protein intake.

## 2. Materials and Methods

### 2.1. Study Participants

This study used data from the prospective, population-based Ansan and Ansung cohorts, which are part of the Korean Genome Epidemiology Study [44]. This data set was provided by the Center for Genome Science, National Institute of Health, Korea Disease Control and Prevention Agency, Republic of Korea. The Ansan–Ansung cohort study is a longitudinal study that investigates the genetic and environmental causes of common metabolic and cardiovascular diseases. The cohort survey was performed biennially until 2012. As the third survey (2005–2006) provided the most detailed dietary information of the participants, we used the third survey as a baseline and included data up to the sixth survey (2011–2012) for analysis.

**Figure 1:**
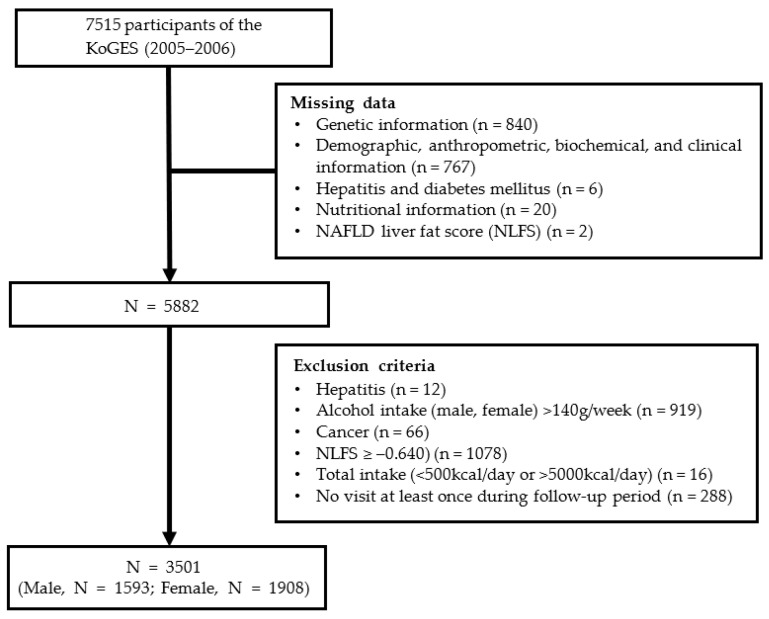
A flow chart of the study population.

During the baseline survey, 7515 people aged 40–69 years living in Ansan and Ansung were enrolled in the study conducted by Korea University and Ajou University. All study participants were informed, and they provided informed consent prior to commencing the study. A total of 4014 participants were excluded before the investigation for the following reasons: (1) lack of information regarding their genetic information (n = 840), clinical data (n = 767), nutritional data (n = 20), hepatitis and diabetes status (n = 6), and NAFLD liver fat score (NLFS)-based diagnosis (n = 2) in the third survey (2005–2006); (2) hepatitis diagnosis (n = 78); (3) alcohol consumption >140 g per week (n = 919); (4) the presence of cancer, including liver cancer (n = 66); (5) NLFS ≥ –0.640 (n = 1080) in the third survey (2005–2006); (6) total caloric consumption <500 or >5000 kcal/day (n = 16); and (7) lack of visit during the follow-up period (n = 288). Finally, 3501 participants were included in this study, as shown in Figure 1. The study was approved by the Institutional Review Board of Ewha Womans University, Seoul, Republic of Korea (IRB approval number: ewha-202105-0003-01).

### 2.2. Demographic, Anthropometric, and Biochemical Data of the Study Population

Data, including age, sex, educational background, physical activity, smoking, drinking, and disease history, were collected via a questionnaire. Body mass index (BMI) was calculated using the equation: weight (kg) divided by the square of the height (m^2^). Waist circumference was measured at the middle area between the ribs and iliac crest, and the average of triplicate measurements was determined. Blood pressure was measured once from the right arm in a sitting position.

Blood samples were obtained from fasting participants to measure fasting glucose and insulin, glycated hemoglobin (Hba1c), total cholesterol (TCh), triglyceride (TG), lipoprotein (high-density lipoprotein cholesterol [HDL-C] and low-density lipoprotein cholesterol [LDL-C]), and liver enzyme (aspartate aminotransferase [AST] and alanine aminotransferase [ALT]) levels.

Smoking, diabetes mellitus (DM), and educational status variables were collected as well as frequency and amount of alcohol consumption. Smoking was classified into “never-smoker”, “former smoker”, and “current smoker”. Participants diagnosed with DM were either clinically diagnosed or had Hba1c and plasma glucose levels ≥6.5% and ≥200 mg/dL, respectively, after a 2 h oral glucose tolerance test or a fasting plasma blood glucose level ≥126 mg/dL (https://www.diabetes.or.kr/pro/; accessed on 1 September 2021). Educational status was categorized into “elementary school or below”, “middle school”, “high school”, and “college or above”. Physical activity was evaluated as follows: 0 metabolic equivalent (MET) for no activity, 1.5 MET for motionless activity, 3 MET for light activity, 5 MET for intermediate activity, and 7 MET for strong activity [45].

### 2.3. Dietary Assessment

Dietary intake was evaluated using a semi-quantitative food frequency questionnaire (FFQ) containing 106 food items [46]. In the FFQ, participants recorded the average frequency and amount of intake over the past year. Caloric, protein, carbohydrate, and fat intakes were calculated as percentages and amounts in grams per day using previously calculated individual nutrient intakes. To determine the criteria for low and high protein intakes, protein-intake level was divided into two and three groups based on the Korean recommended nutrient intake (RNI, from the 2020 Korean Dietary Reference Intake [47]) and intake tertiles, respectively.

### 2.4. NAFLD Diagnosis Using the NLFS

Since results from the liver biopsy did not exist in the Ansan–Ansung study data, NAFLD diagnostic criteria was used instead. Among the various NAFLD diagnostic criteria, the NLFS was utilized for NAFLD diagnosis [48]. The following equation was applied:

NLFS = –2.89 + 1.18 × metabolic syndrome (yes = 1/no = 0) + 0.45 × DM (yes = 2/no = 0) + 0.15 × fasting insulin (mU/L) + 0.04 × AST (U/L) − 0.94 × (AST/ALT).

An NLFS value ≥ –0.640 indicated NAFLD.

### 2.5. Quality Control, Genotyping, and Genetic-Variant Selection

Genetic data from the Ansan–Ansung study were obtained using an Affymetrix Genome-wide SNP Array 5.0 (Affymetrix Inc., Santa Clara, CA, USA) [49]. The quality control (QC) exclusion criteria before imputation were as follows: a minor allele frequency (MAF) < 0.01, Hardy-Weinberg equilibrium (HWE) < 10^−6^, cell rate < 95%, non-autosomal SNPs, and SNPs without strand information or genomic position [50]. Imputation was performed using 1000 Genome Imputation Project Phase-1 v3 [51]. The QC exclusion criteria after imputation were as follows: imputed SNPs with an estimated r^2^ (rsq) < 0.3, MAF < 0.01, and HWE < 10^−6^. A total of 6,461,358 variants remained after QC. Before QC, 3 *FGF21* SNPs, 8 *FGFR1* SNPs, 204 *FGFR2* SNPs, 1 *FGFR3* SNP, and 10 *KLB* SNPs were called from the loci encoding FGF21 and its 4 receptors (not shown). FGFR4 reportedly exhibited a weak interaction with FGF21; therefore, it was excluded from the investigation [19]. After QC, 2 *FGF21*, 3 *FGFR1*, 57 *FGFR2*, and 4 *KLB* SNPs remained. Regarding *FGFR3*, no SNP with a value ≥ 0.01 was detected when the MAF cutoff was applied.

### 2.6. PHS Development and Calculation for NAFLD

QC and the PHS were calculated using Plink version 1.9 (https://zzz.bwh.harvard.edu/plink/index.shtml; accessed on 1 September 2021). Before calculating the PHS, forward stepwise analysis was used to determine the best combination that reflected post-QC NAFLD risk. We determined the area under the curve (AUC) of the receiver operating characteristic (ROC) curve via forward stepwise analysis to confirm the SNP combination’s suitability. The AUC of the ROC curve, as calculated using Proc logistic in SAS, was 0.5693. The PHS was calculated as the participant’s genotype for eight selected SNPs and parameter estimates (β) from a Cox proportional hazards regression.

### 2.7. Expression Quantitative Trait Loci (eQTL) Analysis of Eight SNPs Using Genotype-Tissue Expression (GTEx)

eQTL analysis was performed using GTEx Projects (release version 8) [52]. We sought to determine whether the multiple SNP-affected tissues were related to NAFLD.

### 2.8. Statistical Analysis

All statistical analyses were performed using SAS software version 9.4 (SAS Institute, Inc., Cary, NC, USA), except for QC and PHS. Continuous and categorical variables are expressed as frequencies (%) and mean values (± standard deviations). For baseline analyses, the Mann–Whitney Wilcoxon test and chi-squared test were used to compare continuous and categorical variables, respectively. Cox Proportional Hazard Regression (Cox regression) was used to assess NAFLD incidence after adjusting for confounding variables. The probability in regression analysis was adjusted for sex, age, BMI, ALT level, physical activity, smoking status, educational level, hypertension, DM, hyperlipidemia, menopause (only for women), alcohol consumption, and total caloric intake according to previous studies [53,54,55,56]. The interaction between the PHS and NAFLD was examined using Cox regression, including the interaction term and the Wald test. The *p*-values for the trends across protein intake levels (equal to or above/below the RNI and tertiles) were calculated using a multivariable logistic regression model, with protein intake levels as continuous variables. The *p*-values for the trends between the PHS and NAFLD were determined using the generalized linear model after adjusting for the abovementioned confounding factors.

## 3. Results

### 3.1. Baseline Characteristics and Nutritional Intake

Table 1 shows the participants’ characteristics after categorizing them into non-incident (non-NAFLD group) and incident (NAFLD group) NAFLD groups at baseline. Among the 3501 participants, 1521 developed NAFLD within the follow-up period. The mean age was higher in the NAFLD group than in the non-NAFLD group. In terms of physique-related and biochemical information, the NAFLD group exhibited higher fasting blood glucose, insulin, AST, ALT, TCh, TG, LDL-C, and NLFS levels than the non-NAFLD group. Nevertheless, it is noteworthy that the mean biochemical-variable values spanned the normal reference-value range in both groups, except for TG, whose mean slightly exceeded the normal range in both groups (not shown). The number of patients with DM and hyperlipidemia, excluding those with hypertension, was higher in the NAFLD group than in the non-NAFLD group. Lifestyle and dietary habits, exercise, and carbohydrate intake (%) were more pronounced in the NAFLD group than in the non-NAFLD group.

### 3.2. SNP Selection for PHS

To calculate the PHS of the NAFLD-associated FGF21 pathway, 226 SNPs at the loci of FGF21 and its receptors were initially obtained from the *FGF21, FGFR1, FGFR2, FGFR3*, and *KLB* loci. After removing those with MAFs < 0.01, 179 SNPs were retained (Table S1). The composition of the PHS for NAFLD was determined using forward stepwise analysis. A total of eight SNPs (*KLB* rs2608819, *FGFR1* rs881301, and *FGFR2* [rs9420328, rs4751832, rs7913828, rs2420941, rs1649181, and rs17101702]) were selected (Table 2).

**Figure 2:**
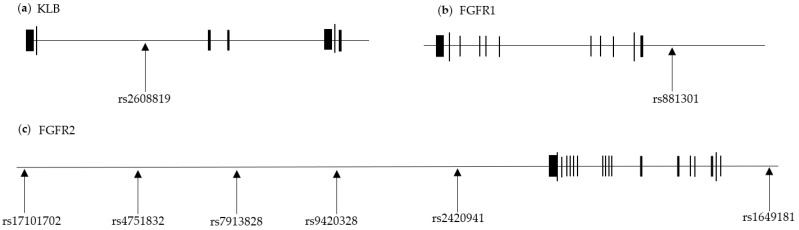
Eight SNPs of FGF21-related loci. The horizontal black lines represent (**a**) KLB, (**b**) FGFR1, and (**c**) FGFR2 loci. The black blocks on the line represent exons. The SNPs used to calculate the PHS are shown their locations in each locus.

The locations of the SNPs relevant to the genes are presented in Figure 2. The AUC value was verified using the ROC curve (AUC = 0.5693). The SNPs’ *β*-adjusted values and risk alleles for NAFLD incidence are shown in Table 2.

### 3.3. Association between the PHS and NAFLD Incidence

We validated the PHS with the rate of NAFLD incidence. Among all participants, those with higher PHS values exhibited significantly higher hazard risks of NAFLD (*p*-trend < 0.0001); a similar trend was observed in male (*p*-trend = 0.0171) and female (*p*-trend < 0.0001) participants, as expected (Table 3).

In women, compared with the first quartile, all other PHS quartiles indicated a significantly higher risk, whereas in men, the third and fourth quartiles, but not the second, exhibited a higher risk. The increased hazard ratio (HR) was relatively greater in women than in men.

### 3.4. Association between the PHS and NAFLD Incidence by Protein Intake

Subsequently, we verified the association between protein intake and NAFLD risk prior to investigating the interaction. Table 4 shows the association between protein intake and the HR for NAFLD when protein intake level was divided into tertiles (low, medium, and high) or into two groups (intake ≥ or <RNI). Protein intake was not significantly associated with NAFLD risk after adjusting for confounding factors. In women, the unadjusted model and model 1 revealed that low protein intake appeared to be associated with the HR for NAFLD; however, the significance of this association disappeared in models 2 and 3.

Furthermore, we sought to determine whether the association between the FGF21-related PHS and NAFLD risk varied with protein intake. The results showed that protein intake modified the association in women only (Table 5). Protein intake affected NAFLD risk in a varied manner depending on the PHS quartile (*p*-interaction = 0.0131 and 0.0361).

In women only, with the lowest PHS values, NAFLD risk was significantly higher in the low-protein-intake group than in the high-protein-intake group (HR = 2.921, *p*-trend <0.0001). In contrast, women with the highest PHS values exhibited a marginally elevated risk in the medium-protein-intake group (HR = 1.435, *p*-trend = 0.0203). When categorizing protein intake based on the RNI, a differential effect of protein intake level was more pronounced. Women with the lowest PHS values had a high risk of NAFLD when consuming a protein level lower than the RNI (HR = 2.021, *p*-trend = 0.0016) compared with those with an intake ≥ the RNI, whereas those with higher PHS values exhibited a high risk, regardless of protein intake level. The results revealed the contribution of FGF21-related genetic variants and restricted protein intake to NAFLD incidence.

### 3.5. Potential Effects of Genetic Variants on Gene Expression

**Figure 3:**
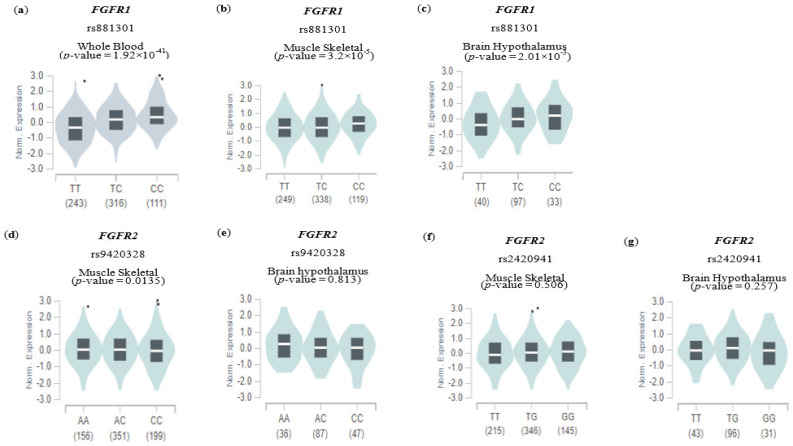
Expression quantitative trace loci (eQTL) analysis of genetic variants. The effect of genetic variables involved in gene expression in other tissues is shown using an eQTL violin plot. Each plot shows the density distribution of each genotype in (**a**–**c**) FGFR1 rs881301, (**d**,**e**) FGFR2 rs9420328, and (**f**,**g**) FGFR2 rs2420941. The white line on the black box plot represents the median value of the expression of each SNP in the genotype. The data were verified using the GTEx Portal website, and the data included tissue-specific information.

eQTL analysis was performed to determine whether the selected SNPs potentially affected gene expression in various tissues. eQTL information for only three of the eight SNPs (*FGFR1* rs881301, *FGFR2* rs9420328, and *FGFR2* rs2420941) were available to demonstrate whether an SNP influences the expression level of one’s corresponding gene in GTEx (Figure 3). Intriguingly, the C allele of FGFR1 rs881301 (an NAFLD risk allele, shown in Table 2) was shown to significantly increase FGFR1 expression in various tissues, with the highest significance occurring in whole blood (*p* value = 1.92 × 10^−41^) and the lowest in musculoskeletal and brain hypothalamus tissues (*p* values = 3.2 × 10^−5^ and 2.01 × 10^−3^, respectively). However, the NAFLD risk alleles *FGFR2* rs9410328 and *FGFR2* rs242041 (C and T alleles, respectively) did not significantly affect the expression of their corresponding genes in musculoskeletal and brain hypothalamus tissues, except for a marginal effect of rs9410328 in musculoskeletal tissue. The results indicate that some of the SNPs, such as FGFR1 rs881301, but not all, may be functionally linked to NAFLD risk via gene-expression alteration.

## 4. Discussion

In this study, we developed a FGF21-related PHS to explore the genetic contribution of the FGF21 pathway to NAFLD incidence and sought to ascertain whether the association between the PHS and NAFLD risk is modulated by dietary protein intake level. A few previous studies have demonstrated the genetic contribution of FGF21 pathways to the metabolic condition. For example, Kaess et al. investigated the association of 63 common SNPs in 5 loci involved in the pathway with metabolic phenotypes, including LDL-C, HDL-C, TG, and BMI, and found FGFR2 polymorphism (rs2071616) to be associated with LDL-C in the European population, a phenomenon that was validated by two other European cohorts [57]. Ji et al. reported that SNPs in the *KLB* gene were correlated with BMI (rs7670903) and hepatic inflammation (rs7674434 and rs12152703) in the Han Chinese population [58]. Although these results suggest that genetic variants linked to the FGF21 pathways are potentially involved in NAFLD pathogenesis, the evidence was based on cross-sectional studies, and the effect size of individual SNPs was limited. In our study, the PHS was developed by selecting 8 out of 226 SNPs at the *FGF21* gene and its receptor genes.

The association between PHS and NAFLD risk was confirmed by showing a positive association with NAFLD incidence in both gender (*p*-trend = 0.0171 and <0.0001 in males and females, respectively, Table 3). The combination of SNPs in the PHS might be related to elevation of the FGF21 pathway. Although how the SNPs impact the risk of NAFLD needs to be further elucidated, GTEx analysis indicated that at least some of the SNPs such as rs881301 at the *FGFR1* locus were significantly associated with upregulation of the corresponding gene in various tissues (Figure 3). Since FGFR1 expression was reported to be positively correlated with FGF21 expression [25,26], the eQTL result was in line with the fact that serum level as well as hepatic expression level of FGF21 were positively associated with the intrahepatic steatosis grade and hepatic triglyceride levels, respectively [25,26,27].

Interestingly, inadequate protein intake (<RNI) compared with adequate intake (≥RNI) in women significantly increased NAFLD risk in participants with the lowest PHS values (HR 2.021) but not in those with the highest PHS values (*p*-interaction = 0.0361, Table 5). These results imply that inadequate protein intake may contribute to NAFLD incidence in people with low genetic risk potentially via FGF21-pathway induction, while those with high genetic risk already have relatively elevated FGF21-pathway activity, regardless of protein intake level. FGF21-pathway stimulation upon protein restriction has been well documented in both human and animal models [34,35,36], exhibiting the upregulation of not only FGF21 but also its receptors. In addition, several GWAS analyses have revealed that gene variants in FGF21 and its receptors are related to diet composition [59,60,61]. *FGF21*-gene variants such as rs838133 and rs838145 are associated with high carbohydrate intake and low protein or fat intake, respectively [59,60]. The results indicate a potential link between the FGF21 pathway and dietary macronutrient distribution. In addition, fructose consumption, which was not accessed in this study, might be a candidate modulator of the association between the FGF21 pathway and the risk of NAFLD. Dietary factors, including fructose consumption, have been extensively studied for their contribution to the risk of NAFLD [62]. Intriguingly, a recent study has indicated that fructose ingestion can stimulate the level of circulating FGF21 [63]. To gain a deeper understanding of this relationship, further studies are required.

The protein-intake-modified association between the PHS and NAFLD risk was more evidently observed in women only. However, the reason for the significant interaction in women remains unclear. It could be due to the sex-differential expression of receptors and response to FGF21. A recent animal study thoroughly investigated the effects of sex and genetic background on metabolic, physiologic, and molecular responses to protein restriction [38]. In fact, FGF21’s response to a low-protein diet was sexually dimorphic. Female mice exhibited a significant gain in fat mass in the low-protein group but no differences in body weight and lean mass. In contrast, male mice displayed dramatic loss of body weight and lean mass but no change in fat mass. FGF21 could be responsible for these metabolic changes. Hormonal changes in women related to conditions such as polycystic ovary syndrome (PCOS) are another potential factor in the development of NAFLD. Recent research, including a meta-analysis of 15 studies, has shown a strong association between PCOS and the risk of NAFLD, independent of BMI [64]. This association has also been confirmed in a study involving Korean women [65]. Since our dataset did not have information about hormonal changes in the participants, further studies are needed to address and minimize the potential bias that might have contribute to the observed difference in women.

This study has certain limitations. First, it was conducted with a limited number of participants from one ethnic population. Genetic association studies are susceptible to population stratification where differences in allele frequency between cases and controls emanate from systematic differences in ancestry. This study’s findings require validation using a larger, independent cohort involving other ethnic populations. In addition, the analysis was limited to a relatively old collection of data. Validation of the findings on recent data will be beneficial. Second, we ascertained participants’ NAFLD statuses using the NFLS, which is a predictive equation for diagnosing NAFLD [48]. Although ultrasound and biopsy are the gold standards for NAFLD diagnosis, the NFLS possesses high sensitivity and specificity, and this was confirmed in the Korean population [66]. Third, PHS development using multiple SNPs in a specific pathway is a promising approach for predicting the risk of complex diseases. It is based on common SNPs in the genes related to the FGF21 pathway accessible from the Affymetrix Genome-wide SNP Array 5.0. However, we cannot exclude the possibility of additional SNPs that were not available on the array but potentially contributed to NAFLD risk. Fourth, we lacked details regarding prescribed drugs that may affect liver health. Finally, we cannot rule out unmeasured or residual confounding variables.

Notwithstanding, to the best of our knowledge, this is the first study to investigate the genetic contribution of the FGF21 pathway to NAFLD risk using the PHS and establish its modification by dietary intake in Korean adults. In conclusion, in women only, genetic variants in the genes encoding FGF21 and its receptors were collectively associated with NAFLD risk. Moreover, protein intake less than the RNI increased NAFLD risk in the participants with the lowest PHS values; however, it did not affect the NAFLD incidence rate in those with higher PHS values. Further investigation is required to validate these findings.

## Figures and Tables

**Table 1:** Study participants’ baseline characteristics according to NAFLD incidence.

Variables in the Third Survey	Non-Incident NAFLD	Incident NAFLD	*p*
Number, n (%)	1980 (56.6)	1521 (43.4)	-
Age (years)	55.0 ± 8.7	56.1 ± 8.6	0.0001
BMI (kg/m^2^)	25.5 ± 3.2	25.3 ± 3.2	0.0916
Waist circumference (cm)	83.9 ± 9.0	83.0 ± 8.6	0.0025
Systolic blood pressure (mmHg)	115.8 ± 17.1	114.2 ± 16.0	0.0076
Diastolic blood pressure (mmHg)	76.9 ± 10.5	76.3 ± 10.0	0.0492
Fasting Blood Glucose (mg/dL)	87.8 ± 8.3	93.3± 13.6	<0.0001
Insulin (µIU/mL)	6.2 ± 2.0	7.4 ± 2.6	<0.0001
Total cholesterol (mg/dL)	188.4 ± 31.7	194.8 ± 34.4	<0.0001
Triglyceride (mg/dL)	107.8 ± 57.7	151.9 ± 103.3	<0.0001
HDL-C (mg/dL)	47.0 ± 10.3	42.3 ± 9.2	<0.0001
LDL-C (mg/dL)	120.0 ± 28.4	122.3 ± 31.0	0.0166
AST (U/L)	23.1 ± 6.7	24.0 ± 6.8	<0.0001
ALT(U/L)	18.4 ± 7.5	21.8 ± 9.4	<0.0001
Alcohol intake (g/week)	17.0 ± 32.6	16.8 ± 32.1	0.4577
Smoking status, n (%)	-	-	0.8330
Never smoker	1266 (64.0)	986 (64.9)	-
Former smoker	354 (17.9)	269 (17.7)	-
Current smoker	359 (18.1)	265 (17.4)	-
Education level, n (%)	-	-	<0.0001
Elementary or below	612 (31.0)	593 (39.4)	-
Junior high school	412 (20.8)	316 (20.8)	-
High school	698 (35.3)	440 (29.0)	-
College or above	254 (12.8)	170 (11.2)	-
Physical activity, METs-hr/wk	335.5 ± 105.8	337.7 ± 110.2	0.6882
NLFS	−1.9 ± 0.7	−1.6 ± 0.6	<0.0001
Diabetes mellitus, n (%)	62 (3.1)	126 (8.3)	<0.0001
Hypertension, n (%)	364 (18.4)	234 (15.4)	0.0194
Hyperlipidemia, n (%)	685 (34.6)	877 (57.7)	<0.0001
Total calorie intake (kcal/day)	1776.8 ± 530.0	1762.7 ± 566.3	0.0749
Protein (g/day)	58.4 ± 22.7	58.3 ± 25.2	0.1218
CHO (g/day)	322.5 ± 88.7	322.2 ± 92.8	0.4375
Fat (g/day)	28.1 ± 17.0	26.7 ± 17.1	0.0005
Protein (%) ^(1)^	12.9 ± 2.3	12.9 ± 2.4	0.3370
CHO (%) ^(1)^	72.5 ± 6.4	73.1 ± 6.5	0.0024
Fat (%) ^(1)^	13.5 ± 5.1	12.8 ± 5.1	<0.0001

*p*-values were calculated by Mann–Whitney Wilcoxon test for continuous variables and chi-squared test for categorical variables. Significance was set at *p* < 0.05. Abbreviations: BMI, body mass index; HDL-C, high density lipoprotein cholesterol; LDL-C, low density lipoprotein cholesterol; AST, aspartate aminotransferase; ALT, alanine aminotransferase; MET-hr/wk, metabolic equivalent of task-hour/week; CHO, carbohydrate; NLFS, non-alcoholic fatty liver disease liver fat score. ^(1)^ Percentages of energy intake from CHO, protein and fat were calculated as follows: CHO intake and protein intake (g/day) × 4 kcal/total energy intake (kcal/day) × 100%, fat intake (g/day) × 9 kcal/total energy intake (kcal/day) × 100%.

**Table 2:** HRs for NAFLD and information of SNPs in PHS.

Gene	SNP ID	CHR	Position (hg19)	Major Allele	Minor Allele	Risk Allele	MAF	HWE	β Adjusted ^(1)^	HR (95%CI) ^(1)^	SE ^(1)^	*p* ^(1)^
KLB	rs2608819	4	chr4:39429811	C	T	T	0.1726	0.6820	0.1184	1.126 (0.840–1.509)	0.1495	0.4286
FGFR1	rs881301	8	chr8:38332318	T	C	C	0.3244	0.9034	0.0976	1.103 (0.929–1.309)	0.0876	0.2653
FGFR2	rs9420328	10	chr10:123140661	A	C	C	0.0996	0.9527	0.2260	1.254 (0.724–2.170)	0.2800	0.4196
	rs4751832	10	chr10:123023263	G	C	G	0.3124	0.5361	0.2047	1.227 (1.024–1.470)	0.0921	0.0263
	rs7913828	10	chr10:123095255	G	A	G	0.3098	0.8229	0.2629	1.301 (1.068–1.583)	0.1003	0.0088
	rs2420941	10	chr10:123229626	T	G	T	0.4338	0.7618	0.1676	1.183 (1.018–1.374)	0.0764	0.0283
	rs1649181	10	chr10:123375856	C	T	C	0.0105	1.0000	0.3501	1.419 (0.922–2.184)	0.2200	0.1115
	rs17101702	10	chr10:123003707	G	C	G	0.1035	0.0016	0.0408	1.042 (0.705–1.539)	0.1991	0.8375

Abbreviation: SNP, single-nucleotide polymorphism; CHR, chromosome; MAF, major allele frequency; HWE, Hardy–Weinberg equilibrium; HR, hazard ratio; 95% CI, 95% confidence interval; SE, standard error; NAFLD, non-alcoholic fatty liver disease; PHS, polygenic hazard score. ^(1)^ Adjusted for sex (male or female), age (years, continuous), BMI (continuous), ALT, physical activity, smoking status (never smoker, former smoker, or current smoker), education level (elementary or below, junior high school, high school, and college or above), diabetes mellitus (yes or no), hypertension (yes or no), hyperlipidemia (yes or no), alcohol intake (g/week, continuous) and total calorie intake (kcal/day, continuous).

**Table 3:** Adjusted HRs for NAFLD according to PHS.

	Cases	HR (95% CI)	*p*-Trend
**All** ^(1)^			
First quartile	864	1.00	**<0.0001**
Second quartile	840	1.280 (1.098–1.488)	
Third quartile	919	1.279 (1.103–1.483)	
Fourth quartile	878	1.572 (1.359–1.819)	
**Male** ^(2)^			
First quartile	396	1.00	**0.0171**
Second quartile	382	1.155 (0.915–1.457)	
Third quartile	412	1.248 (1.002–1.555)	
Fourth quartile	403	1.455 (1.169–1.812)	
**Female** ^(3)^			
First quartile	468	1.00	**<0.0001**
Second quartile	458	1.357 (1.104–1.669)	
Third quartile	491	1.336 (1.093–1.634)	
Fourth quartile	491	1.648 (1.353–2.007)	

^(1)^ Adjusted for sex (male or female), age (years, continuous), BMI (continuous), ALT, physical activity, smoking status (never smoker, former smoker, or current smoker), education level (elementary or below, junior high school, high school, and college or above), diabetes mellitus (yes or no), hypertension (yes or no), hyperlipidemia (yes or no), alcohol intake (g/week, continuous) and total calorie intake (kcal/day, continuous). ^(2)^ Male was adjusted for ^(1)^ except for sex. ^(3)^ Female was adjusted for ^(1)^ plus menopause except for sex.

**Table 4:** Association between protein intake and NAFLD incidence using Cox regression.

	Protein Intake (g/Day)			*p*	*p*-Trend	Protein Intake (g/Day)		*p*	*p*-Trend
	Low	Medium	High			<RNI	≥RNI		
**All** ^(1)^									
unadjusted	1.084 (0.960–1.226)	1.012 (0.894–1.147)	1.00	0.3719	0.1756	1.088 (0.984–1.204)	1.00	0.0992	0.1326
model 1	0.988 (0.867–1.125)	0.990 (0.873–1.124)	1.00	0.9809	0.5572	1.061 (0.957–1.176)	1.00	0.2613	0.1305
model 2	0.935 (0.820–1.066)	0.962 (0.847–1.092)	1.00	0.5996	0.7173	1.026 (0.925–1.138)	1.00	0.6314	0.4391
model 3	0.982 (0.810–1.191)	0.990 (0.850–1.152)	1.00	0.9830	0.8125	1.121 (0.9778–1.285)	1.00	0.0998	0.1066
**Male** ^(2)^									
unadjusted	0.901 (0.748–1.087)	1.010 (0.841–1.213)	1.00	0.4264	0.1116	0.939 (0.807–1.093)	1.00	0.4186	0.2141
model 1	0.932 (0.769–1.131)	1.069 (0.889–1.286)	1.00	0.3775	0.5033	0.971 (0.832–1.133)	1.00	0.7086	0.7556
model 2	0.942 (0.776–1.143)	1.080 (0.898–1.300)	1.00	0.3779	0.4400	0.965 (0.827–1.127)	1.00	0.6569	0.6636
model 3	1.109 (0.830–1.481)	1.186 (0.949–1.483)	1.00	0.2954	0.6973	1.064 (0.864–1.310)	1.00	0.5604	0.5315
**Female** ^(3)^									
unadjusted	1.304 (1.106–1.539)	1.140 (0.962–1.351)	1.00	0.0067	0.0003	1.238 (1.082–1.417)	1.00	0.0019	0.0010
model 1	1.127 (0.948–1.339)	1.064 (0.896–1.263)	1.00	0.3992	0.0331	1.134 (0.988–1.303)	1.00	0.0742	0.0403
model 2	1.049 (0.881–1.249)	1.050 (0.883–1.248)	1.00	0.8273	0.2002	1.069 (0.929–1.230)	1.00	0.3504	0.2388
model 3	1.161 (0.900–1.498)	1.117 (0.908–1.374)	1.00	0.4893	0.1628	1.145 (0.956–1.371)	1.00	0.1417	0.2438

The data were presented as HR (95% CI). ^(1)^ model 1: adjusted for sex, age, BMI, and ALT; model 2: further adjusted for physical activity, smoking status (never smoker, former smoker, or current smoker), education level (elementary or below, junior high school, high school, and college or above), diabetes mellitus, hypertension, hyperlipidemia, and alcohol intake (g/week); and model 3: further adjusted for total calorie intake (kcal/day). ^(2)^ Male was adjusted for ^(1)^ except for sex. ^(3)^ Female was adjusted for ^(1)^ plus menopause except for sex.

**Table 5:** Interaction between the PHS and NAFLD incidence by protein intake using Cox regression.

PHS		Protein Intake (g/Day)			*p*- Trend	*p*- Interaction	Protein Intake (g/Day)		*p*-Trend	*p*- Interaction
	Cases	Low	Medium	High			<RNI	≥RNI		
**All** ^(1)^						**0.0189**				0.8363
First quartile	864	1.244 (0.800–1.934)	0.861 (0.603–1.229)	1.00	**0.0105**	-	1.494 (1.087–2.054)	1.00	**0.0287**	-
Second quartile	840	0.830 (0.559–1.232)	0.908 (0.667–1.237)	1.00	0.6085	-	1.045 (0.788–1.386)	1.00	0.8925	-
Third quartile	919	0.873 (0.603–1.263)	0.843 (0.628–1.132)	1.00	0.4591	-	1.004 (0.774–1.302)	1.00	0.8165	-
Fourth quartile	878	1.062 (0.740–1.524)	1.464 (1.103–1.945)	1.00	**0.0366**	-	1.075 (0.835–1.384)	1.00	0.3938	-
**Male** ^(2)^						0.2071				0.1090
First quartile	396	1.087 (0.571–2.072)	1.125 (0.688–1.840)	1.00	0.8071	-	1.060 (0.668–1.684)	1.00	0.6988	-
Second quartile	382	1.858 (0.964–3.582)	1.794 (1.084–2.970)	1.00	0.1571	-	1.252 (0.794–1.974)	1.00	0.4704	-
Third quartile	412	0.721 (0.402–1.292)	0.823 (0.527–1.286)	1.00	0.6315	-	0.854 (0.575–1.269)	1.00	0.7186	-
Fourth quartile	403	1.529 (0.900–2.598)	1.614 (1.060–2.458)	1.00	0.3049	-	1.397 (0.937–2.083)	1.00	0.1200	-
**Female** ^(3)^						**0.0131**				**0.0361**
First quartile	468	2.921 (1.523–5.602)	1.803 (1.054–3.083)	1.00	**<0.0001**	-	2.021 (1.276–3.200)	1.00	**0.0016**	-
Second quartile	458	0.649 (0.392–1.075)	0.835 (0.553–1.261)	1.00	0.5836	-	0.920 (0.636–1.330)	1.00	0.5114	-
Third quartile	491	1.171 (0.711–1.927)	0.861 (0.571–1.299)	1.00	0.4094	-	1.176 (0.830–1.666)	1.00	0.8531	-
Fourth quartile	491	1.016 (0.627–1.646)	1.435 (0.986–2.089)	1.00	**0.0203**	-	0.877 (0.629–1.222)	1.00	0.4348	-

The data were presented as HR (95% CI). ^(1)^ Adjusted for sex, age, BMI, ALT, physical activity, smoking status (never smoker, former smoker, or current smoker), education level (elementary or below, junior high school, high school, and college or above), diabetes mellitus, hypertension, hyperlipidemia, alcohol intake (g/week), and total calorie intake (kcal/day). ^(2)^ Male was adjusted for ^(1)^ except for sex. ^(3)^ Female was adjusted for ^(1)^ plus menopause except for sex.

## Data Availability

The KoGES data are available upon request from the National Research Institute of Health [37]. The GTEx dataset is available from the NIH dbGAP, study number phs000424.v8.p2.

## Supplementary Materials

The following supporting information can be downloaded at: https://www.mdpi.com/article/10.3390/nu15102385/s1, Table S1: Information of SNPs in FGF21, FGFR1, FGFR2 and KLB genes.
