# Supplementary material for: Association of NAFLD with FGF21 Polygenic Hazard Score, and Its Interaction with Protein Intake Level in Korean Adults
Source: Nutrients. 2023 May 19;15(10):2385. doi: 10.3390/nu15102385 (PMC10220598; doi:10.3390/nu15102385)
Supplement: Supplementary file 1 [file nutrients-15-02385-s001.zip › nutrients-2378642-supplementary.pdf]

Supplementary Table S1. Information of SNPs in FGF21, FGFR1, FGFR2 and KLB genes

Gene	SNP ID	CHR	Position(hg19)	Minor allele	Major allele	MAF	HWE
FGF21	rs599486	19	chr19:49280035	T	C	0.01193	0.63890
	rs11670491	19	chr19:49288662	C	G	0.27580	0.14320
FGFR1	rs2288696	8	chr8:38286225	A	G	0.09519	0.57830
	rs11777067	8	chr8:38298647	T	C	0.34680	0.20490
	rs12677355	8	chr8:38329943	T	C	0.44290	0.07024
	rs881301	8	chr8:38332318	C	T	0.32440	0.90340
	rs13279569	8	chr8:38341616	A	G	0.08552	0.83830
FGFR2	rs2420902	10	chr10:122810409	G	A	0.07279	0.13400
	rs11199734	10	chr10:122819400	A	T	0.39180	0.53210
	rs2420899	10	chr10:122828846	G	T	0.28430	0.89610
	rs11199747	10	chr10:122830043	C	T	0.12270	0.84340
	rs17101395	10	chr10:122837553	A	G	0.38090	0.57290
	rs17101398	10	chr10:122837580	T	A	0.14060	0.86020
	rs11819556	10	chr10:122838775	T	A	0.31700	0.60600
	rs11199756	10	chr10:122841079	A	G	0.38110	0.58840
	rs2252014	10	chr10:122844482	A	G	0.31690	0.60600
	rs2252004	10	chr10:122844709	A	C	0.27840	0.85300
	rs2248282	10	chr10:122852702	G	A	0.06861	0.35970
	rs2247808	10	chr10:122856670	A	T	0.06799	0.31300
	rs1948334	10	chr10:122857317	C	G	0.46860	0.32590
	rs2130790	10	chr10:122864285	A	C	0.40020	0.49230
	rs11199770	10	chr10:122868170	A	G	0.20050	0.38840
	rs17101435	10	chr10:122871745	T	A	0.07048	0.80790
	rs2438673	10	chr10:122872046	A	G	0.45330	0.40260
	rs11199776	10	chr10:122872265	A	G	0.44130	0.89700
	rs2243609	10	chr10:122874348	C	A	0.40440	1.00000
	rs2254079	10	chr10:122875356	C	T	0.16350	0.27640
	rs10444045	10	chr10:122875446	C	T	0.20040	0.33580
	rs2250826	10	chr10:122879441	C	T	0.27530	0.43950
	rs2130779	10	chr10:122879732	T	G	0.07048	0.80790
	rs2250810	10	chr10:122879869	T	C	0.27530	0.43950
	rs2250683	10	chr10:122880681	A	G	0.27530	0.43950
	rs2250682	10	chr10:122880694	A	G	0.27550	0.40890
	rs11199783	10	chr10:122886721	G	C	0.24860	0.17180
	rs1907235	10	chr10:122888555	T	G	0.30150	0.11170
	rs1907236	10	chr10:122888805	C	G	0.20660	0.07975
	rs1907241	10	chr10:122897984	G	T	0.33740	0.84910
	rs10430638	10	chr10:122900306	C	T	0.21040	0.44240
	rs1907243	10	chr10:122900767	T	G	0.21050	0.44240
	rs1907244	10	chr10:122901064	G	A	0.21040	0.44240

rs2253203	10	chr10:122907292	T	C	0.04033	0.27160
rs2451877	10	chr10:122911231	A	G	0.04033	0.27160
rs2130785	10	chr10:122914945	C	G	0.04033	0.27160
rs17101518	10	chr10:122914969	G	A	0.29920	0.64800
rs7904102	10	chr10:122916891	G	C	0.29930	0.64800
rs17101534	10	chr10:122918875	C	T	0.29920	0.62990
rs11199803	10	chr10:122919079	G	A	0.29920	0.62990
rs11199805	10	chr10:122919206	T	C	0.29930	0.64800
rs11199806	10	chr10:122919309	A	G	0.29930	0.64800
rs6585696	10	chr10:122921563	G	A	0.29940	0.66650
rs10886861	10	chr10:122921577	A	C	0.21060	0.46190
rs6585699	10	chr10:122921660	C	T	0.29970	0.72280
rs7906567	10	chr10:122922458	C	A	0.29970	0.72280
rs11199809	10	chr10:122923499	G	A	0.21050	0.46190
rs7897890	10	chr10:122927754	T	C	0.27150	0.40460
rs11199811	10	chr10:122929073	A	C	0.27140	0.38950
rs10886863	10	chr10:122929493	T	C	0.33980	1.00000
rs11199815	10	chr10:122938921	T	C	0.20470	0.74400
rs7085142	10	chr10:122939374	C	T	0.40750	0.11280
rs1907285	10	chr10:122943447	G	A	0.19910	0.07165
rs11199816	10	chr10:122944362	C	T	0.19910	0.07165
rs11199817	10	chr10:122944426	A	G	0.19910	0.07165
rs11199826	10	chr10:122954601	G	C	0.23390	0.21250
rs10788149	10	chr10:122967170	G	A	0.25680	0.34340
rs10749408	10	chr10:122967526	C	T	0.25640	0.38730
rs2172071	10	chr10:122968030	C	T	0.25630	0.37200
rs1907220	10	chr10:122970923	A	G	0.25710	0.30300
rs17101658	10	chr10:122971522	C	T	0.27120	0.85060
rs1907221	10	chr10:122972427	C	T	0.25770	0.27830
rs10510092	10	chr10:122977030	T	G	0.18480	0.37760
rs1907229	10	chr10:122979684	A	G	0.25530	0.73710
rs11199839	10	chr10:122989993	C	T	0.20640	0.43580
rs12220647	10	chr10:122995141	A	C	0.27880	0.59690
rs11199849	10	chr10:122995312	C	T	0.20670	0.34710
rs7922045	10	chr10:123001732	C	T	0.48680	0.41860
rs7899480	10	chr10:123003635	T	C	0.45700	0.40330
rs7899611	10	chr10:123003689	T	C	0.45700	0.41550
rs17101702	10	chr10:123003707	C	G	0.10350	0.00159
rs1873451	10	chr10:123010477	C	T	0.49400	0.56570
rs11199859	10	chr10:123019551	T	C	0.07031	0.14220
rs1125527	10	chr10:123019616	A	G	0.49370	0.45650
rs1125528	10	chr10:123019952	A	T	0.27290	0.08629
rs4319451	10	chr10:123020251	G	A	0.13050	0.28100

rs11199862	10	chr10:123022956	A	G	0.27200	0.22680
rs4751832	10	chr10:123023263	C	G	0.31240	0.53610
rs10788155	10	chr10:123023456	C	G	0.31240	0.53610
rs7923130	10	chr10:123026502	A	G	0.34690	0.42480
rs7922901	10	chr10:123026519	G	C	0.34690	0.42480
rs11199867	10	chr10:123027404	T	G	0.34290	0.46450
rs10886885	10	chr10:123030481	T	G	0.24650	0.88620
rs12247272	10	chr10:123030508	A	G	0.04491	0.90140
rs11199874	10	chr10:123032519	A	G	0.24730	0.75340
rs4468286	10	chr10:123034391	A	C	0.25220	0.86570
rs12413648	10	chr10:123038897	A	G	0.20760	0.49720
rs10788165	10	chr10:123044214	G	T	0.34490	0.55620
rs4609533	10	chr10:123061268	C	T	0.31950	0.24050
rs7097525	10	chr10:123062540	C	T	0.31930	0.26040
rs11199884	10	chr10:123063174	A	G	0.12610	0.17640
rs11199900	10	chr10:123077996	T	A	0.17300	0.33380
rs4752536	10	chr10:123092807	A	G	0.35910	0.40550
rs7913694	10	chr10:123095186	A	G	0.45550	0.63710
rs7913828	10	chr10:123095255	A	G	0.30980	0.82290
rs7895870	10	chr10:123098249	C	T	0.39890	0.67350
rs10886925	10	chr10:123131571	A	G	0.36830	0.32570
rs10886926	10	chr10:123131594	C	T	0.36880	0.30390
rs10886927	10	chr10:123132045	C	T	0.31640	0.76800
rs9420328	10	chr10:123140661	C	A	0.09955	0.95270
rs1896404	10	chr10:123141130	A	T	0.33250	0.70150
rs2420929	10	chr10:123153176	T	C	0.46490	0.35800
rs17101921	10	chr10:123153295	A	G	0.23360	0.90540
rs9421422	10	chr10:123154188	G	C	0.08614	1.00000
rs4457689	10	chr10:123176129	G	A	0.37080	0.52330
rs4751840	10	chr10:123179594	G	A	0.29490	0.66380
rs1896413	10	chr10:123181038	G	A	0.14030	0.92970
rs733521	10	chr10:123183873	T	G	0.29460	0.29410
rs10788178	10	chr10:123184875	G	A	0.28920	0.60490
rs2420926	10	chr10:123188264	C	T	0.29150	0.47100
rs11199964	10	chr10:123193998	C	T	0.43310	0.74530
rs10886937	10	chr10:123195615	A	G	0.25880	0.97790
rs3948460	10	chr10:123198212	A	T	0.01951	0.77660
rs7911028	10	chr10:123198286	T	G	0.26180	0.56340
rs2162543	10	chr10:123198332	T	C	0.23260	0.27040
rs6585734	10	chr10:123198490	T	C	0.26180	0.56340
rs2114691	10	chr10:123198836	T	C	0.18530	0.14870
rs17096089	10	chr10:123198960	A	C	0.21210	0.72640
rs7918550	10	chr10:123205203	C	T	0.06606	0.86310

rs7902280	10	chr10:123205281	A	G	0.06601	0.73020
rs10788183	10	chr10:123205357	T	C	0.18500	0.27410
rs11199974	10	chr10:123214477	G	T	0.04525	0.21790
rs7074862	10	chr10:123216849	G	C	0.46140	0.34640
rs12776781	10	chr10:123218381	G	C	0.28500	0.54830
rs1007359	10	chr10:123218471	A	G	0.01171	0.63560
rs7911727	10	chr10:123224879	A	G	0.21270	0.84890
rs1619436	10	chr10:123226944	T	C	0.24340	0.95390
rs2420941	10	chr10:123229626	G	T	0.43380	0.76180
rs10749418	10	chr10:123235061	G	T	0.44850	0.41390
rs1649202	10	chr10:123240625	A	G	0.42780	0.10320
rs2278202	10	chr10:123243197	A	G	0.41690	0.16810
rs1078806	10	chr10:123338975	G	A	0.24840	0.30520
rs3135715	10	chr10:123354726	G	T	0.27760	0.28890
rs1649181	10	chr10:123375856	T	C	0.01052	1.00000
rs1696813	10	chr10:123377197	C	A	0.24260	1.00000
rs1219636	10	chr10:123384845	G	A	0.01041	1.00000
rs10788190	10	chr10:123387029	G	A	0.24500	0.90840
rs3104685	10	chr10:123391364	C	G	0.18670	0.83360
rs10466158	10	chr10:123399847	C	T	0.14410	0.69790
rs11200052	10	chr10:123402237	T	C	0.09915	0.67710
rs7917459	10	chr10:123402374	C	T	0.20000	0.48530
rs10749424	10	chr10:123405155	G	T	0.44650	0.21200
rs10788194	10	chr10:123406645	G	A	0.44650	0.25410
rs9971131	10	chr10:123431515	T	G	0.45460	0.60670
rs10510099	10	chr10:123431846	A	C	0.11450	0.75290
rs3104688	10	chr10:123436465	T	G	0.17020	0.25890
rs1693688	10	chr10:123436696	G	T	0.27340	0.91470
rs1696803	10	chr10:123436950	T	C	0.17040	0.29200
rs1696853	10	chr10:123437207	G	A	0.47100	0.98300
rs2935718	10	chr10:123437453	T	C	0.47100	0.98300
rs1693687	10	chr10:123438113	T	C	0.47090	1.00000
rs17102407	10	chr10:123441100	G	A	0.24800	0.88670
rs7086628	10	chr10:123442841	C	G	0.43540	0.98270
rs1219505	10	chr10:123460906	T	C	0.23610	0.61630
rs1219508	10	chr10:123461654	T	C	0.04972	0.57440
rs1693682	10	chr10:123463733	A	G	0.38710	0.39430
rs11200107	10	chr10:123488039	T	C	0.22750	0.50560
rs11200108	10	chr10:123488067	C	T	0.05543	0.61110
rs2935706	10	chr10:123491668	C	G	0.27880	0.29020
rs753455	10	chr10:123494644	C	T	0.29460	0.66360
rs752736	10	chr10:123495655	G	C	0.46440	0.76470
rs11200120	10	chr10:123498426	C	T	0.47780	0.78170

KLB	rs900563	4	chr4:39409416	A	C	0.35970	0.01741
	rs11940694	4	chr4:39414993	G	A	0.40270	0.47930
	rs2608819	4	chr4:39431431	T	C	0.17260	0.68200
	rs6854452	4	chr4:39446337	A	T	0.39280	0.18830
	rs1458254	4	chr4:39446807	T	C	0.05396	0.17590
	rs2687971	4	chr4:39452944	G	C	0.44900	0.66730
	rs2687972	4	chr4:39453668	G	A	0.44910	0.73090
	rs2608828	4	chr4:39453730	G	C	0.29390	0.95910
	rs10517522	4	chr4:39453790	G	A	0.39280	0.40940
	rs2608829	4	chr4:39453730	A	G	0.44920	0.74710
